# Human Enterovirus Group B Viruses Rely on Vimentin Dynamics for Efficient Processing of Viral Nonstructural Proteins

**DOI:** 10.1128/JVI.01393-19

**Published:** 2020-01-06

**Authors:** Paula Turkki, Mira Laajala, Malin Flodström-Tullberg, Varpu Marjomäki

**Affiliations:** a Department of Biological and Environmental Science, Division of Cell and Molecular Biology/Nanoscience Center, University of Jyväskylä, Jyväskylä, Finland; b Faculty of Medicine and Health Technology and BioMediTech, Tampere University, Tampere, Finland; c Center for Infectious Medicine, Department of Medicine Huddinge, Karolinska Institute, Karolinska University Hospital, Stockholm, Sweden; Instituto de Biotecnologia/UNAM

**Keywords:** apoptosis, enterovirus, polyprotein processing, proteases, vimentin

## Abstract

We report that several viruses from the human enterovirus group B cause massive vimentin rearrangements during lytic infection. Comprehensive studies suggested that viral protein synthesis was triggering the vimentin rearrangements. Blocking the host cell vimentin dynamics with β, β′-iminodipropionitrile (IDPN) did not significantly affect the production of progeny viruses and only moderately lowered the synthesis of structural proteins such as VP1. In contrast, the synthesis of the nonstructural proteins 2A, 3C, and 3D was drastically lowered. This led to attenuation of the cleavage of the host cell substrates PABP and G3BP1 and reduced caspase activation, leading to prolonged cell survival. Furthermore, the localization of the proteins differed in the infected cells. Capsid protein VP1 was found diffusely around the cytoplasm, whereas 2A and 3D followed vimentin distribution. Based on protein blotting, smaller amounts of nonstructural proteins did not result from proteasomal degradation but from lower synthesis without intact vimentin cage structure. In contrast, inhibition of Hsp90 chaperone activity, which regulates P1 maturation, lowered the amount of VP1 but had less effect on 2A. The results suggest that the vimentin dynamics regulate viral nonstructural protein synthesis while having less effect on structural protein synthesis or overall infection efficiency. The results presented here shed new light on differential fate of structural and nonstructural proteins of enteroviruses, having consequences on host cell survival.

**IMPORTANCE** A virus needs the host cell in order to replicate and produce new progeny viruses. For this, the virus takes over the host cell and modifies it to become a factory for viral proteins. Irrespective of the specific virus family, these proteins can be divided into structural and nonstructural proteins. Structural proteins are the building blocks for the new progeny virions, whereas the nonstructural proteins orchestrate the takeover of the host cell and its functions. Here, we have shown a mechanism that viruses exploit in order to regulate the host cell. We show that viral protein synthesis induces vimentin cages, which promote production of specific viral proteins that eventually control apoptosis and host cell death. This study specifies vimentin as the key regulator of these events and indicates that viral proteins have different fates in the cells depending on their association with vimentin cages.

## Abstract (precis)

A virus needs the host cell in order to replicate and produce new progeny viruses. For this, the virus takes over the host cell and modifies it to become a factory for viral proteins. Irrespective of the specific virus family, these proteins can be divided into structural and nonstructural proteins. Structural proteins are the building blocks for the new progeny virions, whereas the nonstructural proteins orchestrate the takeover of the host cell and its functions. Here, we have shown a mechanism that viruses exploit in order to regulate the host cell. We show that viral protein synthesis induces vimentin cages, which promote production of specific viral proteins that eventually control apoptosis and host cell death. This study specifies vimentin as the key regulator of these events and indicates that viral proteins have different fates in the cells depending on their association with vimentin cages.

## INTRODUCTION

Human enteroviruses (EVs) are a large group of viruses including rhinoviruses, echoviruses, group A and B coxsackieviruses, and polioviruses. They are among the most common viruses infecting humans worldwide. Most commonly, EVs cause acute infections, leading to lytic cell death with rapid clearance of the virus by the immune system (1). However, in some cells, infection can become persistent and lead to chronic infection (2). Deciphering the cellular events during viral infection is the key to understanding the consequences and pathology of virus infections.

Enteroviruses have four structural (VP1 to VP4) proteins that form the icosahedral virus capsid and ten nonstructural (2A, 2B, 2C, 2BC, 3A, 3B, 3AB, 3C, 3D, and 3CD) proteins, with several different functions. Enteroviral protease 2A cleaves the cellular eukaryotic translation initiation factor 4 G (eIF4G) and poly(A) binding protein (PABP), controls apoptosis, and induces stress granule formation (3–6). Protease 3C cleaves the cellular Ras GTPase-activating protein-binding protein 1 (G3BP1) and PABP (4, 6, 7). Protein 3D is an RNA-dependent polymerase and has been shown to be involved in the inflammatory response via the activation of NLRP3 inflammasome (8). All of the viral proteins are processed from a single polyprotein, and viral protein processing has been shown to be cellular chaperone mediated (9–11).

Vimentin is the most common intermediate filament in several cell types. Its expression is altered during development and in certain diseases. Vimentin has a high degree of similarity among species, suggesting that it plays a vital role in normal cellular functions. Several research groups have reported the spatial association of vimentin with viruses during infection, particularly near the replication area and progeny virus production (12–34). Despite the abundance of such reports, there is still no consensus on the role played by vimentin during virus infections. In addition, the mechanisms by which the virus triggers vimentin remodeling remains undefined. In addition to virus infections, vimentin is associated with several significant human diseases. During cancer development, vimentin expression correlates with tumor growth, invasiveness, and poor prognosis. In addition to its structural role, vimentin has been shown to function as a key regulator of organelle positioning (35), cell migration, adhesion, and cell signaling (36).

In our earlier studies, we noticed that the morphology of the cellular vimentin network correlated with echovirus-1 (EV-1) infection efficiency in tested human cell lines (14). Changes in the vimentin network brought about with different media and treatments correlated with successful baculovirus transduction and echovirus infection, suggesting that the vimentin network has a previously unknown role in infection. Here, we hypothesized that, in highly permissive cells, virus could modify the vimentin network for its own benefits, most likely via cellular stress processes that it has been shown to regulate. Here, we have tested this hypothesis with careful monitoring of the cellular vimentin network and several vimentin-related stress responses throughout EV infection.

We show that infection by a member of the human EV group B viruses leads to massive rearrangements of the intermediate filament, vimentin. When vimentin dynamics are inhibited, expression of the viral nonstructural proteins is affected, the cellular targets of 2A and 3C, PABP and G3BP1, remain uncleaved, and cell death is postponed. In contrast, VP1 expression is only slightly decreased and infective progeny viruses are produced. Our data here suggest that the vimentin network plays a regulatory role in viral nonstructural protein expression, contributing to host cell survival, whereas the soluble pool of structural proteins remains largely unaffected by vimentin dynamics.

## RESULTS

### Human EV infection induces drastic vimentin rearrangements that start appearing by the time of replication.

**FIG 1:**
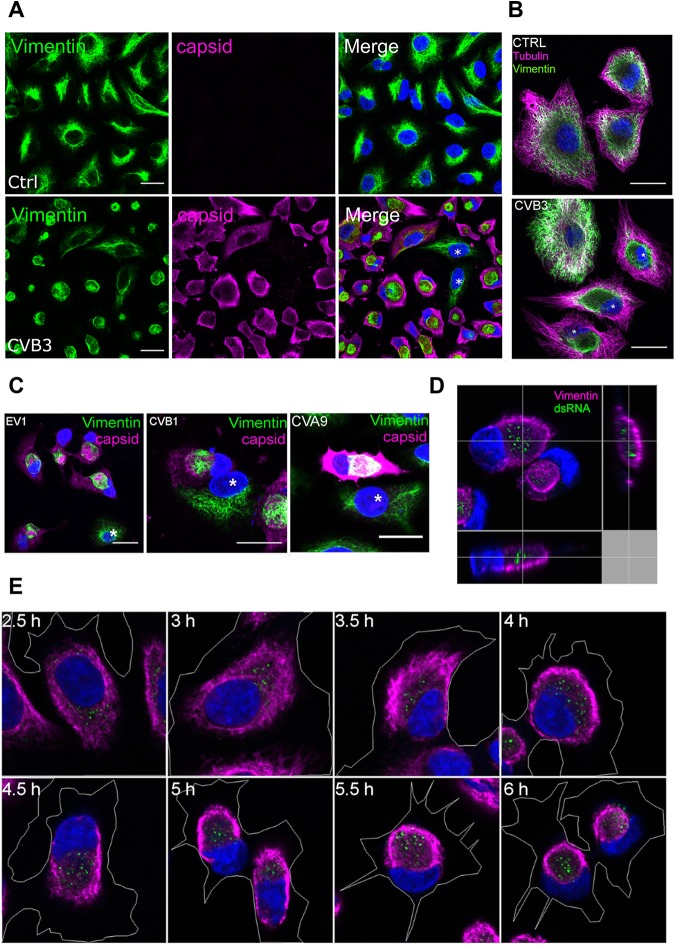
Human enterovirus infection induces vimentin-enwrapped dsRNA-harboring compartment to the perinuclear location in A549 cells. A549 cells were fixed, immunolabeled, and visualized with confocal microscopy. (A) Images from single sections showing vimentin (green) and virus capsid (magenta) in cells after CVB3 (5 h p.i.) infection. (B) Vimentin (green) and tubulin (magenta) network in noninfected (ctrl) and CVB3-infected (5 h p.i.) cells. Infected cells are marked with asterisks. (C) Images from single sections showing vimentin (green) and virus capsid (magenta) in cells after EV1 (6 h p.i.), CVB1 (6 h p.i.), and CVA9 (5 h p.i.) infections. Noninfected cells are marked with asterisks. (D) Projection of Z-sections showing dsRNA (green) and vimentin (magenta). Orthogonal sections providing a view of these structures in three dimensions after CVB3 infection (5 h p.i.). (E) Images of single sections showing vimentin structure formation from 2.5 h to 6 h p.i. Cell boundaries were drawn to visualize the state of cell detachment.

In order to determine the role of vimentin during EV infection, A549 cells were infected with coxsackievirus B3 (CVB3), fixed at different time points postinfection (p.i.), and immunolabeled for virus progeny capsids (VP1) and vimentin. When the composition of the vimentin network was thoroughly analyzed using confocal microscopy, it was noticed that at later stages of infection, when the cytoplasm was full of newly synthetized capsid proteins (4 to 6 h postinfection [p.i.]), the majority of the infected cells showed drastic vimentin rearrangements, leading to the formation of a compact vimentin cage next to the nucleus (Fig. 1A). Furthermore, tubulin labeling was done in order to ensure that the whole cytoskeleton was not affected in infected cells (Fig. 1B). Cells infected with EV1, coxsackievirus B1 (CVB1), and coxsackievirus A9 (CVA9) showed similar vimentin rearrangements in the late stages of their life cycle (Fig. 1C). The vimentin modifications were only seen in infected cells, indicating that these were virus induced (Fig. 1A and C; uninfected cells are shown by asterisks). Capsid protein VP1 was diffusely scattered all around the cytoplasm and on the cell edges, whereas the virus-induced vimentin structure was compact and formed in the perinuclear area (Fig. 1A and C).

We next looked at the association between the replication intermediate double-stranded RNA (dsRNA) and vimentin using an antibody against dsRNA to mark the cells positive for virus replication. It was noticed that vimentin formed a compartment that surrounded the dsRNA (Fig. 1D). A time course study showed that dsRNA and vimentin rearrangements both appeared around 3 to 4 h p.i. and became more pronounced during the progression of infection (Fig. 1E). Vimentin rearrangements started by first forming thicker filaments in the periphery of the cell, leaving the perinuclear area, where dsRNA can usually be seen, devoid of vimentin. As the signal for dsRNA slowly increased, a thick vimentin “barrier” started to decrease in diameter, and eventually, around 5 h p.i., it became a round compartment that contained dsRNA within. However, even if vimentin was accumulating in the perinuclear area, it did not drastically change the cell size or overall morphology, which was visible from the cell outlines marked in the images (Fig. 1E). As cells were still attached to the coverslips, these images verify that the vimentin structures were not formed simply due to cell rounding and detachment.

**FIG 2:**
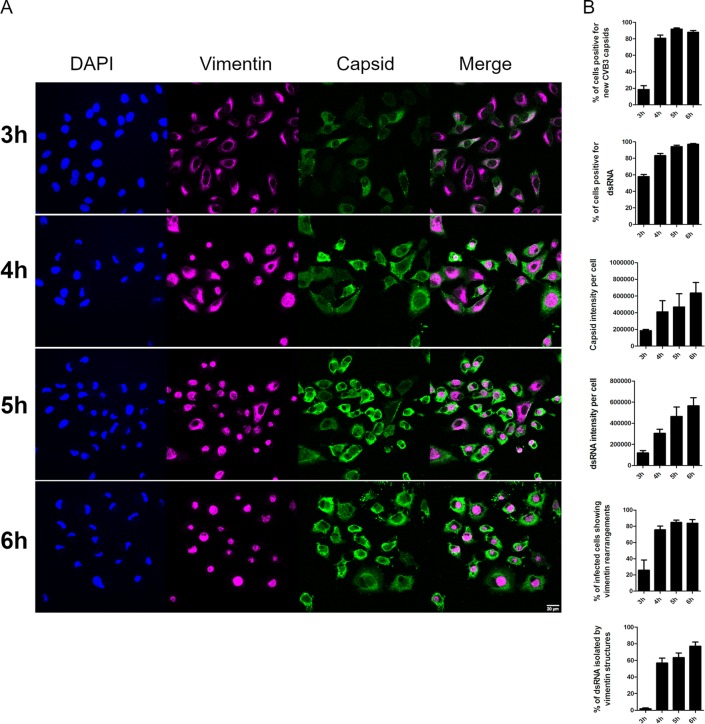
Appearances of VP1 and dsRNA coincide with vimentin rearrangements during CVB3 infection. A549 cells were fixed, immunolabeled, and visualized with confocal microscopy. (A) Single-section images showing vimentin and VP1 (capsid) at different time points p.i. (B) Quantifications of confocal images taken at different time points during CVB3 infection. The results shown here are representations of at least three independent experiments. For the quantifications, approximately 200 cells altogether from two to three replicates were analyzed (±SEM). Scale bars, 20 μm.

We then set out to quantify the relative amounts of cells positive for capsid protein production, to evaluate the intensity of VP1 label in the cells, to quantify the number of infected cells showing virus-induced vimentin compartments and cells positive for viral replication (dsRNA), to measure the intensity of dsRNA label in the cells, and to assess the frequency of dsRNA enwrapped by the vimentin structure during the later time points (3 to 6 h p.i.) (Fig. 2A and B). Altogether, the results showed that at 3 h p.i. around 20% of the cells were positive for newly synthetized VP1 and 60% were positive for dsRNA. However, both the dsRNA and capsid levels per cell were still extremely low, indicating that the replication had just started. From the cells positive for progeny virus production, only 20% showed the typical virus-induced vimentin rearrangements at 3 h p.i. However, as the relative amount of capsids per infected cell started to increase after 4 h p.i., so did the appearance of virus-induced vimentin structures, leading to almost 80% of the infected cells with vimentin compartments surrounding dsRNA. It was clear from the quantification that both dsRNA appearance and capsid protein synthesis started before the virus-induced vimentin compartments started appearing. This suggests that vimentin structure formation is not needed to initiate virus replication. Instead, the emergence of dsRNA or viral proteins could act as a trigger for the vimentin rearrangements to take place.

### Vimentin dynamics are triggered by the emergence of viral proteins.

We then set out to define the trigger for the virus-induced changes in vimentin distribution and structure. To determine whether virus internalization was sufficient or whether later stages of the virus life cycle, such as uncoating and/or replication, were needed for the virus-induced vimentin rearrangements to take place, two approaches, neutral red viruses and UV-inactivated viruses, were used.

**FIG 3:**
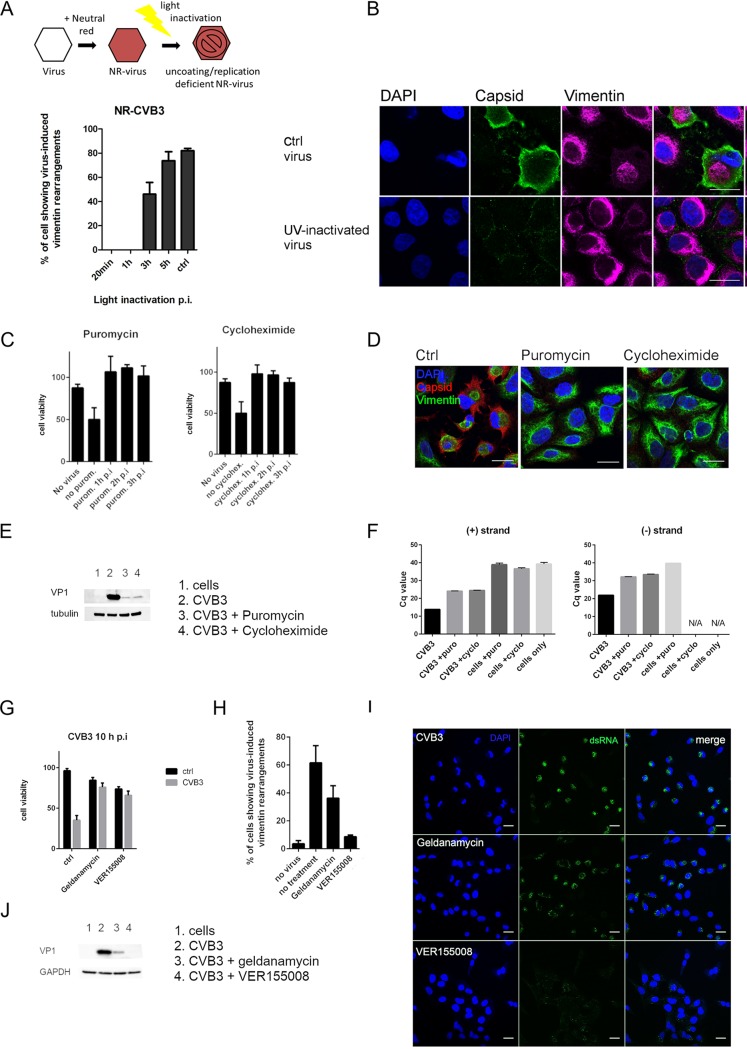
Viral protein synthesis is essential for vimentin cage formation. (A, top) Schematic illustration showing the principle of neutral red viruses. A549 cells were infected with neutral red-CVB3 exposed to light treatment at different time points, and the presence of virus-induced vimentin structures was visualized from single-section confocal images and quantitated. For the quantifications, approximately 50 cells per sample from three replicates were analyzed. (B) Single-section confocal images illustrating the effect of UV-inactivated EV1 on A549 cells at 6 h p.i. Cells were immunolabeled for capsid (green) and vimentin (magenta). (C) Graphs showing the results of the CPE experiment in CVB3-infected A549 cells after differential treatments with either cycloheximide (200 μg/ml) or puromycin (100 μg/ml). Drugs were added to the cells at different time points p.i. and left until the end of the experiment (8 h p.i.). Control cells were normalized to 100%. Data are representative of at least two separate experiments with three replicate samples from each. (D) Single-section confocal images visualizing vimentin (green) and virus capsids (red) in CVB3-infected (5 h p.i.) A549 cells with ctrl, puromycin (100 μg/ml), or cycloheximide (200 μg/ml) treatment when the drugs were introduced at 2 h p.i. Scale bars, 20 μm. Results are representative of at least two separate experiments. (E) Western blot showing VP1 expression in infected cells after cycloheximide (200 μg/ml) or puromycin (100 μg/ml) treatments. The drugs were added at 2 h p.i. and left until the end of the experiment (5.5 h p.i.). Results are representative of at least two separate experiments. (F) RT-qPCR from CVB3-infected cells left untreated or treated with cycloheximide (200 μg/ml) or puromycin (100 μg/ml). Virus (8.86 × 10^7^ PFU/ml) was bound on cells on ice for 1 h. After washing excess virus away, the infection was allowed to proceed for 5.5 h. The drugs were added at 2 h p.i. and left until the end of the experiment. N/A, signal is below detection threshold; *C_q_*, quantification cycle. (G) Graph showing the results of the cell viability measurement (ATP) of CVB3-infected A549 after differential treatments with either VER-155008 (50 μM) or geldanamycin (0.1 μM). Drugs were added to the cells together with the virus and left until the end of the experiment (10 h). Results are representative of at least two separate experiments with three replicate samples from each. (H) The quantification of confocal images of CVB3-infected and VER-155008- and geldanamycin-treated A549 cells showing virus-induced vimentin structures. Data were obtained from at least 100 cells from two independent experiments. (I) Single-section confocal images showing dsRNA (green) in CVB3-infected cells with or without VER-155008 (50 μM) or geldanamycin (0.1 μM) treatment. Virus (4.43 × 10^8^ PFU/ml) was bound on ice for 1 h, and after washing excess virus away, the infection was allowed to proceed for 5.5 h. The drugs were added after ice binding and left until the end of the experiment. Scale bars, 20 μm. (J) Western blot showing VP1 expression in infected cells after VER-155008 (50 μM) or geldanamycin (0.1 μM) treatment. The drugs were added to the cells together with the virus and left until the end of the experiment (5.5 h p.i.). Results are representative of at least two separate experiments.

First, we tested neutral red-labeled CVB3 viruses (NR-CVB3), which are photosensitive and can be light inactivated, resulting in uncoating-deficient viruses (Fig. 3A). Cells infected with NR-CVB3 were either kept in the dark (ctrl) or exposed to light at different time points p.i. After ten minutes of light treatment at room temperature, the cells were incubated at 37°C until 5 h p.i., after which cells were fixed and immunolabeled for virus capsid and vimentin. These results showed that photoinactivated NR-CVB3 viruses were not able to induce the vimentin rearrangements if the inactivation was performed prior to 3 h p.i., i.e., before replication had taken place. When the light inactivation was performed from 3 h p.i. onwards, virus-induced vimentin structures started appearing (Fig. 3A). Light inactivation itself did not alter the vimentin network. Light inactivation at 0 h p.i. totally prevented virus infection, as determined by endpoint titration, confirming that the light inactivation was working correctly (data not shown). Furthermore, NR-CVB3 kept in the dark showed high infectivity (2.18 × 10^8^ PFU/ml), also confirming the functionality of the NR virus.

In addition to the NR-CVB3 experiment, the effects of UV-inactivated EV1 (UV-EV1) viruses were tested (Fig. 3B). Cells were infected either with the wild-type EV1 or with the UV-inactivated EV1, fixed at 5 h p.i., and immunolabeled to visualize EV1 capsids and vimentin. As the results show, UV-inactivated viruses were not able to cause the typical virus-induced vimentin structures that can be seen surrounding the viral dsRNA in infected control cells. Thus, these results suggested that mere internalization and intracellular/endosomal presence of virus is not enough to trigger the vimentin changes.

We next determined whether the genome itself could act as a trigger for the vimentin rearrangements or whether replication and/or protein synthesis was needed. We tested the effects of cycloheximide and puromycin on cells, which earlier were shown to inhibit poliovirus protein synthesis (37). Our results showed that these treatments prevented virus-induced cytopathic effect (CPE) (Fig. 3C), vimentin rearrangements (Fig. 3D), and CVB3 infection, as determined by VP1 expression (Fig. 3E). We also confirmed an efficient inhibition of replication by quantifying the negative- and positive-strand synthesis by quantitative PCR (qPCR) (Fig. 3F). In order to arrest replication by other means, we tested guanidine hydrochloride (GuHCl). GuHCl has been shown to inhibit enteroviral 2C protein, leading to inhibition of the initiation of negative-strand RNA synthesis (38–40). Our results showed that addition of 2 mM GuHCl in early infection completely inhibited virus infection and protein production as detected by immunolabeling of VP1 protein (data not shown). Subsequently, vimentin cages did not form. Although the inhibitor should not impair translation *per se*, it understandably has consequences on silencing infection in general due to the block of replication. To further study the role of replicating dsRNA, we transfected the cells with low and high concentrations of the dsRNA analog poly(I:C) and monitored vimentin dynamics. The results showed that transfection of poly(I:C) into cells did not cause vimentin rearrangements (data not shown). This suggests that the cellular machinery recognizing foreign dsRNA does not trigger the events leading to vimentin dynamics during infection.

Heat shock proteins (Hsps), and Hsp70 in particular, have been associated with several virus infections, such as rabies (41) and dengue (42). Hsp90 was previously shown to be essential for the viral assembly and capsid production of enterovirus 71 (43) and poliovirus (44) by protecting the viral components from proteasomal degradation. Here, we wanted to determine whether Hsp70 and Hsp90 had any role in vimentin dynamics during infection. To accomplish this, we used the specific inhibitor of Hsp70, VER-155008, which is known to bind to the ATP-binding site of Hsp70 and to prevent substrate binding and chaperone activity. In addition, we used the Hsp90 inhibitor geldanamycin. Hsp70 and Hsp90 work in collaboration in cells so that Hsp90 receives its client proteins from Hsp70 in a partially folded state. Although proteins from the Hsp family are also associated with cellular stress and survival, the inhibitors used here act only on the chaperone activity. First, we monitored the cell viability in response to VER-155008 and geldanamycin. Both Hsp inhibitors were able to postpone virus-induced cell death, while VER-155008 was more potent in its effect (Fig. 3G). In addition to preventing cell death, these inhibitors blocked or decreased the vimentin cage formation (Fig. 3H). This also correlated with the decrease of infectivity in total, as determined by dsRNA appearance in the infected cell cytoplasm (Fig. 3I) and VP1 expression in the cells (Fig. 3J).

Altogether, these results indicate that viral protein synthesis is dependent on functional chaperones, especially Hsp70, and that viral protein expression is essential for the vimentin structures to form.

### Inhibiting vimentin dynamics delays host cell death while allowing efficient infection.

**FIG 4:**
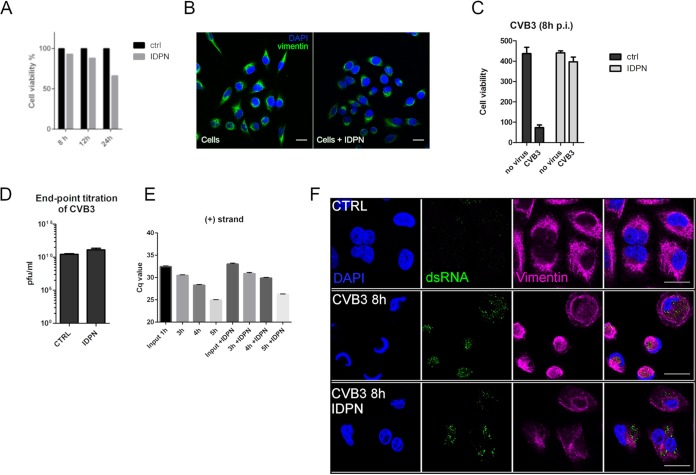
IDPN treatment delays virus-induced cell death without compromising the production of progeny viruses. (A) Graph showing the effect of IDPN treatment on A549 cell viability. Results are representative of two replicates. (B) Single-section images showing vimentin distribution after 5.5 h of 1.5% IDPN treatment. Results are representative of at least three separate experiments. (C) Graph showing cell viability (ATP) in CVB3-infected A549 cells with and without IDPN treatment (1.5%). Drug was added together with the virus and kept until the end of the experiment. Results are representative of at least two separate experiments with three replicate samples from each. (D) Endpoint titration of progeny viruses produced after 6 h of CVB3 infection in A549 cells with or without IDPN treatment. Results are representative of two independent experiments. (E) RT-qPCR from cells infected with CVB3 for 1, 3, 4, or 5 h with or without IDPN treatment. Virus (8.86 × 10^7^ PFU/ml) was bound on cells on ice for 1 h. After washing excess virus away, the infection was allowed to proceed for the indicated time. IDPN was added after ice binding and left until the end of the experiment. (F) Single-section confocal images illustrating the effect of IDPN on replication (dsRNA, green) and vimentin (magenta). Representative image of at least three replicates. Scale bar, 20 μm.

Vimentin is the most common intermediate filament, but there is a shortage of drugs and treatments that can be used to modify its functions. In previous vimentin-related publications, acrylamide and calyculin A have been used to inhibit vimentin dynamics, but in our experiments with A549 cells, the recommended concentrations of these compounds led to rapid cell death (data not shown). We were also unsuccessful in completely knocking down vimentin using a short interfering RNA (siRNA) approach (data not shown). Another drug that has been shown to lead to the disruption of vimentin is β, β′-iminodipropionitrile (IDPN) (45). IDPN was found to be gentle enough to cause only a slight decrease in cell viability during our experimental setup in A549 cells (Fig. 4A). In addition, IDPN treatment did not induce any vimentin changes by itself (Fig. 4B). Remarkably, cells infected in the presence of IDPN were not showing signs of virus-induced CPE, and cell viability remained high even 8 h p.i., whereas in control infection, already over 80% of the infected cells had died (Fig. 4C). Strikingly, this did not correlate with progeny virus production as, indeed, IDPN-treated cells efficiently produced infective virions, similar to control cells, as was judged by endpoint titration (Fig. 4D). Also, only a slight decrease in replication was observed, based on the measurement of positive-strand synthesis using qPCR in IDPN-treated cells (Fig. 4E). This was also confirmed by labeling of dsRNA (Fig. 4F). IDPN did, however, have a clear effect on the localization of dsRNA, as it spread out to a wider area in the cytoplasm from the perinuclear area when formation of vimentin cages was prevented with IDPN.

### The locations of viral nonstructural proteins 3D and 2A follow the location of vimentin in the cells, whereas VP1 localization was not affected by changes in vimentin.

**FIG 5:**
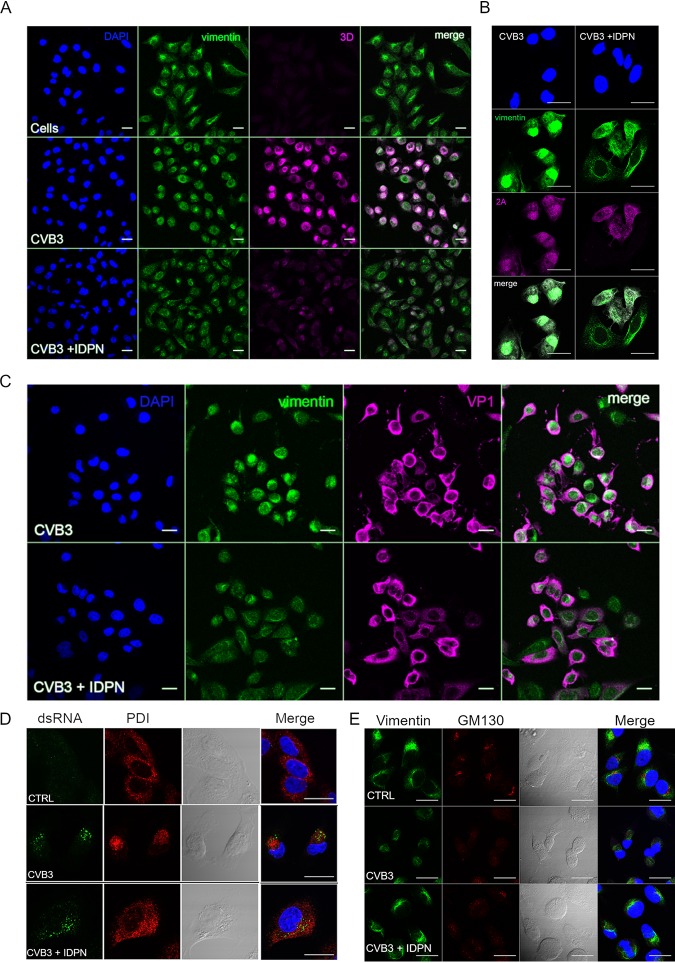
Vimentin cage preferentially hosts nonstructural proteins. (A and B) Single sections showing location of 3D (magenta) (A) or 2A (magenta) (B) in the perinuclear area and vimentin (green) in control or CVB3-infected cells with or without IDPN treatment. Virus (4.43 × 10^8^ PFU/ml) was bound on ice for 1 h. After washing the excess virus away, infection was allowed to proceed for 5.5 h. IDPN was added after ice binding and left until the end of the experiment. (C) Single sections showing the location of VP1 diffusely in the cytoplasm in CVB3-infected cells with or without IDPN treatment. Infection was carried out as described for panel B. Scale bars, 20 μm. Representative images from at least three separate experiments are shown. (D and E) Single-section confocal images illustrating the effect of IDPN on ER (PDI) (5.5. h p.i) (D) and Golgi (GM130) (5.5 h p.i) (E). Scale bar, 20 μm. The images are representative of at least two separate experiments.

We were then curious to monitor the expression of individual viral structural and nonstructural proteins. Confocal microscopy of 3D polymerase showed a notable decrease in 3D expression under IDPN treatment compared to that of normal infection (Fig. 5A). In addition, the localization of the 3D signal was quite well associated with vimentin cages, whereas during IDPN treatment, the signal was spread out in the cell, similar to vimentin label. In addition to 3D, 2A protease showed a similar phenomenon (Fig. 5B). It associated more strongly with the vimentin cage during infection but spread out to all cytoplasm, showing lower signal during IDPN treatment (Fig. 5B). In contrast to these results with nonstructural proteins, VP1 label seemed to be similarly strong during normal infection and IDPN treatment (Fig. 5C). Also, there was no apparent shift in the location of the signal due to IDPN treatment, suggesting that the structural and nonstructural proteins are differentially located during their translation in the cytoplasm with respect to vimentin distribution. This led us to evaluate the location of other cellular components and whether their location would be sensitive to IDPN treatment. Indeed, the luminal endoplasmic reticulum (ER) marker PDI was spread out during IDPN treatment, while during infection it was drawn to the vimentin cage area, colocalizing with dsRNA (Fig. 5D). The *cis*-Golgi matrix protein GM130 was also found to redistribute from the typical perinuclear Golgi location toward vimentin-organized cages (Fig. 5E). This process was partially prevented by IDPN treatment (Fig. 5E).

### Inhibition of vimentin dynamics leads to a marked decrease in nonstructural protein expression compared to viral structural proteins.

**FIG 6:**
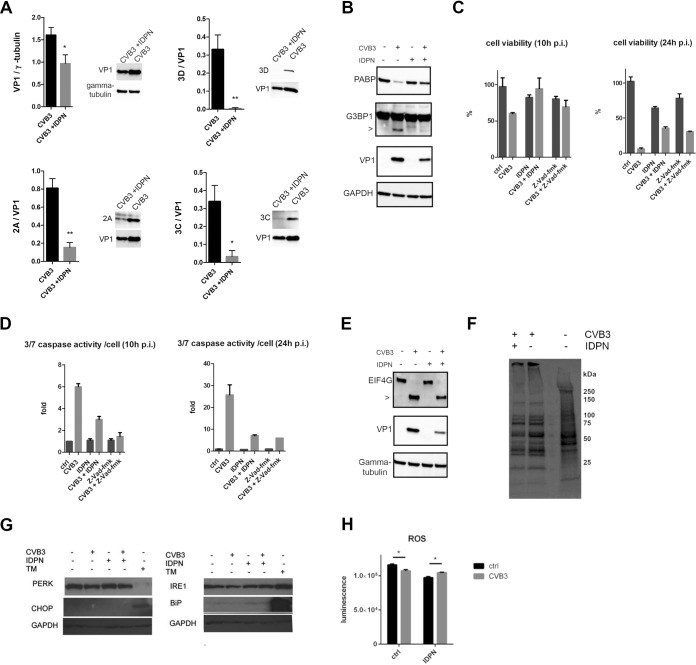
Inhibition of vimentin dynamics affects the levels and activity of nonstructural proteins. (A) Graphs showing quantifications of Western blots where levels of VP1, 2A, 3C, and 3D were detected in CVB3-infected A549 cells with or without IDPN treatment. Virus (4.43 × 10^8^ PFU/ml) was bound on ice for 1 h. After washing the excess virus away, infection was allowed to proceed for 5.5 h. IDPN was added after ice binding and left until the end of the experiment. Band intensities were quantified using Image J, and the quantifications were done from at least three separate experiments. (B) Western blots were immunolabeled with antibodies against PABP, G3BP1, VP1, and GAPDH. The arrowhead indicates CVB3 induced cleavage product. Results are representative of at least two separate experiments. (C and D) Graphs showing the results of the viability measurement (C) and caspase activity (D) per viable cell of A549 cells treated with Z-VAD-fmk (200 μM) or IDPN (1.5%) with or without CVB3 infection. Drugs were added to the cells together with the virus and left until the end of the experiment (10 and 24 h p.i.). Graphs show results from three independent experiments. (E) Western blots were immunolabeled with antibodies against eIF4G, VP1, and GAPDH. Results are representative of at least two separate experiments. (F) Pulse labeling of CVB3-infected cells with or without 1.5% IDPN treatment. Virus (4.43 × 10^8^ PFU/ml) was bound on ice for 1 h, after which the excess virus was washed away. IDPN was added after ice binding and left until the end of the experiment. Pulse labeling with radioactive sulfur (500 μCi/ml) was carried out at 4.5 to 5.5 h p.i. Results are representative of two separate experiments. (G) Immunoblotting performed after SDS-PAGE showing the expression status of different ER markers with and without CVB3 (5.5 h p.i.) and/or IDPN in A549 cells. Tunicamycin (TM; 5 μg/ml) was used as a positive control and GAPDH as a loading control. (H) Luminescence measurement indicating the ROS activation in A549 cells without CVB3 (6 h p.i.) and/or IDPN. Graphs show results from three independent experiments. *, *P* < 0.05.

As the confocal microscopy suggested a clear difference between the expression and location of viral capsid proteins during IDPN treatment compared to that of 3D polymerase and 2A protease, we set out to quantify the amounts of VP1 and different viral nonstructural proteins. First of all, we observed that VP1 expression was about 40% lower than that during normal infection (Fig. 6A). This was in line with the decrease seen in positive-strand synthesis (Fig. 4E). In contrast, the signals for 2A, 3C, and 3D were much lower when evaluated by Western blotting (Fig. 6A). Quantification of all these nonstructural proteins compared to VP1 detected in the same blots revealed that all signals from nonstructural proteins were markedly lower than that of VP1, by only about approximately 20%, 10%, and 1% for 2A, 3C, and 3D, respectively, of the amount of VP1 (Fig. 6A). This decrease coincided well with the lower activity of viral proteases 2A and 3C toward some of their cellular substrates (Fig. 6B). The cellular substrates PABP and G3BP1 were left largely uncleaved despite the infection taking place, also leading to higher cell viability (Fig. 6C). As these substrates have been linked to promotion of apoptosis during infection, we wanted to measure the effects of caspase activation. Indeed, the lower activity toward PABP and G3BP1 coincided with a marked decrease in caspase activation (Fig. 6D), further explaining the lack of CPE in IDPN-treated infected cells.

Interestingly, the cellular substrate of 2A, elF4G, was rather efficiently cleaved, albeit with lower efficiency than the control infection (Fig. 6E). As elF4G is linked to host cell shutoff during viral infection, we evaluated the overall status of protein translation using metabolic labeling and observed a clear host cell shutoff both during normal infection and IDPN treatment (Fig. 6F). Thus, it seems that the minor effect of IDPN on elF4G via 2A still allowed a rather efficient host cell shutoff and efficient production of viral structural proteins during IDPN treatment.

Cell killing during virus infection may also occur via ER stress. To rule out that the prolonged viability and lower cell killing during IDPN treatment had to do with ER stress response, we set out to monitor different ER stress markers and their expression (Fig. 6G). Tunicamycin treatment (24 h) was used as a positive control. CVB3-infected cells with or without IDPN treatment did not show any similarities with tunicamycin treatment or changes in any of these marker proteins, indicating that ER stress was not induced in CVB3-mediated cell death (Fig. 6G). Reactive oxygen species (ROS) have also been associated with vimentin changes in the cells during stressful conditions. However, as we looked at the H_2_O_2_ induction in the cells with the aid of the ROS-Glo kit (Promega), we could only observe minor changes in CVB3 treated cells compared to the control cells either with or without IDPN treatment (Fig. 6H).

These results altogether suggest that when vimentin dynamics are inhibited, cell killing is postponed due to low expression and activity of the nonstructural viral proteases 2A and 3C and not via ER stress or ROS production.

### Inhibiting vimentin dynamics slows down synthesis, especially of nonstructural proteins, but does not accelerate degradation.

**FIG 7:**
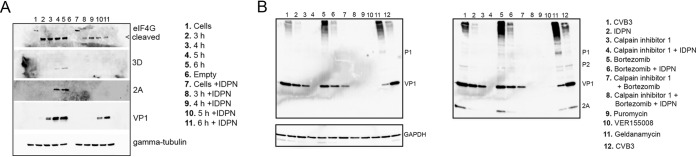
Vimentin dynamics affect the synthesis of nonstructural proteins rather than their degradation. (A) Western blot of A549 cells infected with CVB3 for 3, 4, 5, or 6 h with or without IDPN treatment. Virus (4.43 × 10^8^ PFU/ml) was bound on ice for 1 h. After washing the excess virus away, infection was allowed to proceed for the indicated time. IDPN was added after ice binding and left until the end of the experiment. eIF4G, 3D, 2A, and VP1 were visualized using antibodies against the proteins. Shown is a representative image of two replicates. (B) Western blot showing the effect of 1.5% IDPN, 7 μM bortezomib, 200 μM calpain inhibitor 1, 100 μg/ml puromycin, 50 μM VER-155008, or 0.1 μM geldanamycin on CVB3 infection. Virus (4.43 × 10^8^ PFU/ml) was bound on ice for 1 h. After washing the excess virus away, infection was allowed to proceed for 5.5 h. Other drugs were added after ice binding except calpain inhibitor, bortezomib, and puromycin, which were added at 2 h p.i. All drugs were left until the end of the experiment. Visualization of VP1 and P1 is on the left. Merged image of 2A and VP1 labeling is on the right. Results are representative of at least two separate experiments.

According to our results, the smaller amount of nonstructural proteins seemed to be a key aspect mediating the prolonged viability and reduced cell killing during IDPN treatment. Our results further indicated that during IDPN treatment there is also a marked reduction in nonstructural protein expression versus that of structural proteins. Therefore, a crucial question to be addressed was whether the nonstructural proteins are actively downregulated or inefficiently synthetized or processed. EV polyprotein is synthetized as one unit that is then cleaved and processed into the individual structural and nonstructural proteins. We first set out to define whether smaller amounts of nonstructural proteins are due to active degradation of those proteins. Western blotting and immunostaining of viral proteins were performed from samples taken at different time points during infection, with and without IDPN (Fig. 7A). The results showed that during normal infection the nonstructural proteins 2A and 3D became visible after 4 and 5 h p.i., while VP1 was evident earlier, starting from 3 h p.i. IDPN treatment caused lower synthesis of the VP1 and a delay in the appearance of VP1. In the same blot, 2A and 3D remained undetectable throughout the infection period. As proteasomal degradation is the main mechanism to get rid of cytoplasmic proteins, we first used the specific proteasomal inhibitor bortezomib to assess the levels of VP1 and 2A during viral infection with and without IDPN. The Western blotting results first of all confirmed our earlier observation that VP1 was moderately downregulated during IDPN treatment, whereas 2A was almost nondetectable after 5.5 h p.i. (Fig. 7B, blot on the right, lanes 1 and 2). Addition of bortezomib together with IDPN did not restore normal levels of VP1 or 2A, whereas they stayed similar to those with IDPN treatment alone, suggesting that the lower expression was not due to proteasomal degradation (Fig. 7B, lanes 2, 5, and 6). This result was also confirmed with another proteasomal inhibitor, lactacystin (data not shown).

We also tested the involvement of cytoplasmic neutral proteases, calpains. Calpains are ubiquitous proteases readily available in the cytoplasm and shown by us and other to be involved in promoting enterovirus infections (46–48). Addition of calpain inhibitor 1 around 2 h p.i. caused a more pronounced inhibition on VP1 than mere IDPN treatment (Fig. 7B, lanes 2 and 3). Addition of calpain inhibitor 1 on top of IDPN treatment totally abolished viral protein production and infection (Fig. 7B, lane 4). Our recent results have shown that calpain proteases can contribute to efficient cleavage and maturation of structural proteins from the P1 region of the polyprotein (M. Laajala, M. M. Hankaniemi, J. Määttä, V. P. Hytönen, O. H. Laitinen, and V. Marjomäki, unpublished data). Therefore, the additive effect of calpains with IDPN to totally block both structural and nonstructural proteins was expected.

Western blot results also confirmed that the chaperone Hsp70 inhibitor VER-155008 almost completely shuts down viral protein synthesis (Fig. 7B, lane 10). The Hsp90 inhibitor geldanamycin, on the other hand, had almost an opposite effect for viral protein synthesis compared to that after IDPN treatment: nonstructural protein 2A was expressed in larger amounts than in IDPN treatment, whereas VP1 was found in smaller amounts (Fig. 7B, lanes 2 and 11).

The results altogether confirmed that the changes in vimentin cage formation cause a much higher reduction in synthesis of nonstructural proteins than structural proteins. The results further show that the effect is not executed via increased degradation of viral proteins.

## DISCUSSION

Several viruses have been shown to cause changes in the cellular vimentin network during infection. There are several postulations on the role of vimentin dynamics, but no consensus has been found so far for the mechanisms of action. Vimentin aggregating or collapsing to make a perinuclear compartment has been previously reported with the closely related viruses enterovirus 71 and foot-and-mouth disease virus (20) but also for less closely related viruses, such as vaccinia virus (18), iridovirus (25), bluetongue virus (23), parvovirus (30, 31), African swine fever virus (34), Epstein-Bar virus (19), and dengue virus (16, 29, 32, 49, 50). These aggregates have been shown to surround the replicating DNA (18), dsRNA (16), and nonstructural or newly synthetized structural proteins (16, 20, 23, 29, 32), leading the authors to suggest that vimentin acts to surround the replication and assembly sites and to have a scaffolding or a protective role. Similarly, in our studies, the hallmark of these virus-induced vimentin structures was the cage formation to surround the replication-intermediate dsRNA. However, as vimentin rearrangements also led to ER and Golgi rearrangements, it could be postulated that the dsRNA was concentrating inside these vimentin structures by the redistribution of the ER and Golgi membrane, which provides membranes for the replication processes. In fact, when the formation of these vimentin structures was inhibited, replication and progeny virus production continued, but dsRNA, nonstructural proteins, and ER were more diffusely located around the cell. Translocation of the ER also has been previously reported for dengue virus infection (16). Although the presence of dsRNA or other replication elements within these structures was a constant feature in previously published studies, our results here show that the clustering of replication-associated structures inside the vimentin cage is not a necessary factor for infection and production of progeny viruses.

Our studies show that the formation of vimentin structures was dependent on viral protein translation based on several lines of evidence. (i) Cage formation was inhibited when either UV-inactivated or light-inactivated (neutral red-treated) replication-incompetent viruses were used. (ii) The structures were not seen when cells were transfected with a dsRNA analog or when infected cells were treated with protein synthesis inhibitors. (iii) Finally, the appearance of the structures coincided with the time of viral protein synthesis and could be inhibited by perturbing the function of Hsp70, which efficiently blocked viral protein synthesis (9). Taking this into consideration, we were surprised to see that none of the ER stress markers were upregulated during infection.

Vimentin has been previously shown to protect hepatitis C virus core protein and the cellular protein Scrib from host-mediated proteasomal degradation (15, 51). Proteasomal degradation of hepatitis C virus core protein was inhibited by MG-132, an inhibitor of proteasomal and calpain degradation. In our experiments, MG-132 efficiently inhibited virus infection (data not shown) because of the strong dependence of enterovirus infection on calpain proteases (48 and Laajala et al., unpublished). MG-132 is a strong inhibitor of calpains; therefore, in our study, more specific inhibitors of proteasomal degradation were used, e.g., bortezomib and lactacystin. Those studies showed clearly that proteasomal degradation was not involved in IDPN-induced effects.

In addition to rapid life cycle and clear cytopathic effect, the ability to cease host cell protein synthesis also is a hallmark of enterovirus infection. Enteroviral host cell shutoff and the onset of host cell apoptosis have been linked to the actions of the viral proteases 2A and 3C. Enteroviruses commonly have three viral proteases, which are in charge of viral polyprotein processing and cleavage of cellular targets (52). Protease 3CD is involved in the cleavage of P1, leading to the maturation of the capsid proteins VP1, VP2, and VP0. Pro 2A is believed to autocatalytically cleave P1 from P2, while 3C and 3CD are supposed to take care of other polyprotein cleavages. In our results, we could observe small amounts of 3D, 3C, and 2A expression with IDPN treatment by immunoblotting and immunofluorescence staining. Still, VP1 was observed in rather large amounts and, surprisingly, normal amounts of infectious particles were generated during IDPN treatment. We have unpublished information that calpain proteases 1 and 2 are able to correctly cleave capsid proteins from P1 (Laajala et al., unpublished). Thus, the ubiquitously present calpain proteases in the cytoplasm could contribute to capsid protein processing and explain the almost normal amounts of VP1 during IDPN treatment with lower 3C and 3CD expression. It seems that the small amount of 2A observed during IDPN treatment is enough to efficiently execute the cleavage of P1 out of P2-P3. Also, a small amount of 3D polymerase was observed, which clearly produced enough RNA for the assembly of infectious viral particles.

The cellular targets of 2A and 3C, PABP and G3BP1, are partially responsible for the host cell shutoff. Therefore, it was a surprise that, despite their small amounts, virus infection was accompanied by a rather efficient host cell shutoff. From the cellular targets, the eIF4G cleavage was the least affected, perhaps being responsible for the strong reduction of host cell protein production. During IDPN treatment, ample RNA and structural proteins were still produced during the first 6 h of infection. Still, the high virus yields were somewhat unexpected because of the detected smaller amounts of nonstructural proteins. However, it is likely that at later time points the virus yields are bound to get lower. Rather than affecting the virus yields or host cell shutoff, the more important consequence of the lowered synthesis of 2A and 3C/3CD was the reduced caspase activation. Caspase 3/7 activation was clearly compromised, leading to higher viability, while the viral protein and RNA production continued at an almost normal pace.

Our findings show that human enterovirus infection leads to massive vimentin rearrangements that harbor the replication site, as was indicated by the higher association of dsRNA, 2A, and 3D polymerase with the vimentin cages. Many RNA viruses, including enteroviruses, have been shown to cause massive membrane rearrangements in the host cell during replication. The formation of these replication organelles has been shown to be caused by viral nonstructural proteins such as 3A (53). The replication organelles appear first as single-membrane tubular structures that evolve into double-membrane vesicles, which serve as platforms for replication (54, 55). Since both the time of appearance and localization into perinuclear area coincides with replication organelles and vimentin cage (54, 55), vimentin is likely to have a role in the formation or support of the replication area. In addition, when the vimentin dynamics were prevented, the replication area was more spread out, further suggesting that vimentin contributes to the organization of the replication area. Moreover, it can be speculated that the sequestration of replication area into vimentin cage protects the virus from, e.g., pattern recognition receptors (PRRs) of the host. These PRRs are part of the innate immune system and protect the host from pathogens by recognizing foreign molecules such as dsRNA (56). However, whether the vimentin cage protects enteroviruses against innate immune response of the host cell remains to be shown.

Importantly, we also observed that the replication sites did not particularly accumulate structural proteins such as VP1, which was widely distributed around the cell and, thus, was less affected by the IDPN treatment. Instead, perturbation of these structures reduced the synthesis levels of 2A, 3C, and 3D and processing rather than their selective degradation. Thus, the results indicated that, due to IDPN treatment, cleavage products of P1 and P2 (VP1 and 2A, respectively) were produced in different ratios. It has been shown that the processing of P1 out from the polyprotein occurs cotranslationally, when 2A rapidly cleaves between itself and VP1 as soon as the required components have been translated (57, 58). In light of our results, the synthesis of the rest of the polyprotein (P2-P3) may be dependent on vimentin dynamics and takes place efficiently only if vimentin is specifically arranged. However, it will be important to study the true mechanistic basis behind these phenomena in the future. Speculatively, one explanation could be the various noncanonical translation pathways that RNA viruses use to translate a multitude of proteins from their compact mRNA (59). Many RNA viruses use noncanonical translation, such as ribosomal frameshifting, in order to regulate the ratios of different viral proteins, most commonly allowing greater production of structural proteins (60, 61); among these viruses are also cardiovirus and FMDV from the picornavirus family (62, 63). Whether such mechanisms are contributing to the observed different ratios of nonstructural and structural protein synthesis and processing for CVB3 as well remains to be shown.

**FIG 8:**
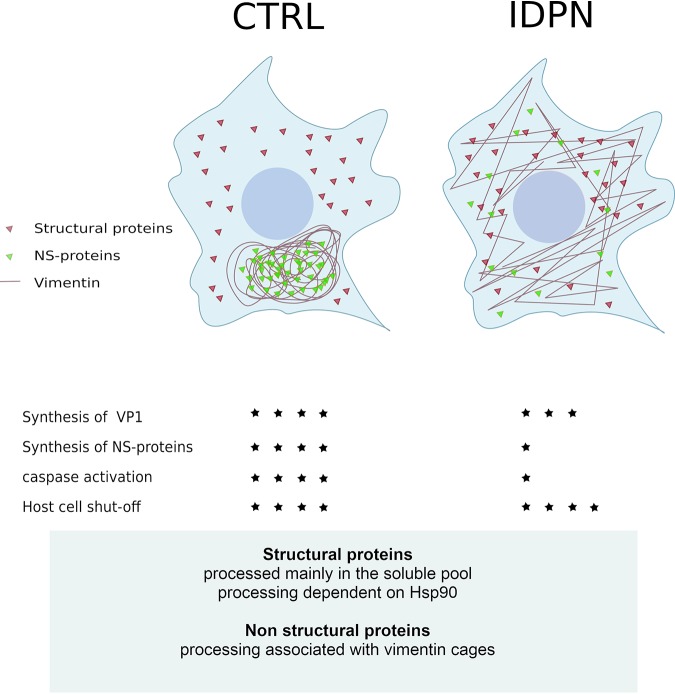
Summary. Viral protein synthesis during enterovirus infection induces formation of a vimentin-enwrapped perinuclear compartment harboring the viral nonstructural proteins. Inhibition of vimentin rearrangements leads to scattered distribution of nonstructural proteins and their lower expression and activity without affecting the structural proteins and viral progeny production. Stars indicate the magnitude of the phenomenon. NS-proteins, nonstructural proteins.

Interestingly, the Hsp90 inhibitor geldanamycin caused an arrest in VP1 production, while the effect in nonstructural proteins was much milder. Hsp90 is known to bind P1 and contribute to P1 processing (11, 44). Thus, results with Hsp90 also suggest that different cellular mechanisms affect P1 and production of structural proteins, in contrast to nonstructural proteins. Vimentin has been shown to coimmunoprecipitate 2C of the foot-and-mouth disease virus, and together they organize replication sites for efficient infection (20). Influenza A virus viral ribonucleoprotein was also shown to be bound by vimentin in the cytoplasm, thereby preventing it from entering the nucleus and rather downregulating the infection (64). Interestingly, Lawson and Semler (65) showed, using metabolic labeling of poliovirus 1, that much of the P1 and structural proteins accumulate in the cytosolic soluble fraction, although P1 also stays partially membrane bound. In contrast, most of the nonstructural proteins, as well as P2 and P3, associate with the membrane-bound fraction, supposedly the replication structures. Their results suggested that P2 and P3 processing is active early in infection *in vivo* in the membranous fraction but does not occur anymore when P2, 3CD, and P3 later appear in the soluble fraction. In contrast, P1 is actively processed further in the soluble fraction for longer periods. These results suggest that the distribution of P2-P3 and their individual proteins in the soluble cytosolic or membrane-bound fraction largely determines their activity in polyprotein processing (Fig. 8). It seems likely that the vimentin cage organizes the replication structures together with 2C and provides an optimal niche for the initial replication/translation to occur and to produce viral proteases 2A, 3C, and 3CD, as well as 3D polymerase. Without cage formation, the replication area is less organized, and the synthesis of nonstructural proteins is less efficient while VP1 production occurs almost normally in the soluble fraction. However, it will be important to study which factors trigger vimentin rearrangements and also reveal the molecular mechanism behind the cage formation. Although we showed the effect of vimentin rearrangements specifically during the infection of enterovirus B species, it is likely that other enterovirus species (A, C, and D) also would show similar dependence on vimentin rearrangements, taking into account the similarity of replication processes among different species.

In conclusion, we show that viral protein synthesis during enterovirus infection induces formation of a vimentin-enwrapped perinuclear compartment harboring replicating dsRNA and nonstructural proteins 3D and 2A. In turn, inhibition of vimentin rearrangements leads to scattered distribution of nonstructural proteins and their lower expression and activity. This leads to delayed onset of apoptosis and higher viability of the host cells. In contrast, location and expression level of structural proteins, such as VP1, stays largely unchanged, promoting efficient virus production. Altogether these results show that vimentin dynamics, taking place in the infected cells, regulate nonstructural protein synthesis without compromising infection efficiency but affecting host cell survival.

## MATERIALS AND METHODS

### Cells.

Human alveolar basal epithelial cell line A549 and human cervix adenocarcinoma cell line HeLa MZ were used for the experiments. The cell lines were obtained from the American Type Cell Culture (ATCC) and grown in humidified 5% (vol/vol) CO_2_ at 37°C in Dulbecco’s modified Eagle’s medium (DMEM; Invitrogen) and 5% to 10% fetal bovine serum (FBS) supplemented with GlutaMAX (Invitrogen) and penicillin and streptomycin (P/S).

### Viruses.

EV1 (Farouk strain), CVA9 (Griggs strain), CVB1 (Conn5 strain), and CVB3 (Nancy strain) were obtained from the ATCC and propagated in green monkey kidney (GMK) cells. The virus was released from infected GMKs by freeze-thawing and concentrated by centrifugation into a sucrose cushion. Infectivity of the produced virus stock was assayed with an endpoint titration, and viruses were used in excess in order to guarantee efficient infection (multiplicity of infection of 65) in A549 cells. When ice binding was used, the number of PFU per milliliter is mentioned for each experiment. For all infection studies, the culture medium was supplemented with 1% to 5% FBS.

### Endpoint dilution.

The assay was carried out in GMK cells (ATCC) cultured in a 96-well plate. Cells were infected with CVB3 by preparing a dilution series in MEM supplemented with 1% FBS and 1% GlutaMAX. After 3 days of infection at 37°C, the cells were stained for 10 min with 50 μl of crystal violet stain (8.3 mM crystal violet, 45 mM CaCl_2_, 10% ethanol, 18.5% formalin, and 35 mM Tris base). The excess stain was washed with water, and the infectivity was determined based on the number of dyed (noninfected) and nondyed (infected) wells. The 50% tissue culture infective dose (TCID_50_) was calculated by comparing the number of infected and uninfected wells for eight replicates of the same virus concentration. The concentration at which half of the wells would be infected was extrapolated (TCID_50_). Finally, the TCID_50_ value was multiplied by 0.7 to obtain the PFU-per-milliliter value. Endpoint dilution for NR-CVB3 was done after inactivating the virus with light for 10 min or keeping the virus in the dark as a control.

### Reagents.

Cycloheximide, puromycin, tunicamycin, and VER-155008 were purchased from Sigma-Aldricht, whereas the caspase inhibitor Z-VAD-fmk, Caspase-Glo 3/7 assay kit, and ROS activity and CellTiter-Glo cell viability kit were obtained from Promega. Other reagents included annexin V (Abcam), IDPN (Alfa Aesar), staurosporine (Enzo), calpain inhibitor I (Roche), geldanamycin (Enzo), bortezomib (LC Laboratories), and GuHCl (Sigma).

### Immunolabeling.

In all immunofluorescence and confocal microscopy studies, the cells were grown on coverslips and fixed with 3% to 4% paraformaldehyde (PFA)–phosphate-buffered saline (PBS). Permeabilization, when needed, was performed with 0.1 to 0.2% Triton X-100–PBS. All used antibodies were diluted in 3% BSA–PBS, and cells were stained by using a standard protocol for immunofluorescence staining with appropriate antibodies. Fluorescence-conjugated goat secondary antibodies against mouse or rabbit antibodies were from Life Technologies. The coverslips were mounted with ProLong Gold antifade reagent with 4′,6-diamidino-2-phenylindole (DAPI; Life Technologies).

### SDS-PAGE and Western blotting.

Cell lysates were suspended in Laemmli buffer containing mercaptoethanol. Samples were separated in 12% SDS-polyacrylamide gel or in 4 to 20% Mini-Protean TGX Stain-Free gel (Bio-Rad) and electroblotted into polyvinylidene difluoride membrane (Millipore). Appropriate primary antibodies together with horseradish peroxidase-conjugated secondary antibodies were used in immunoblotting. Bands were detected by a SuperSignal chemiluminescence detection kit (Thermo Scientific) and developed into X-ray film or imaged with ChemiDoc MP (Bio-Rad).

### Antibodies.

To detect CVA9, CVB1, and CVB3, either polyclonal rabbit antiserum against CVA and CVB (kindly provided by Merja Roivainen, National institute of Health and Welfare, Helsinki, Finland) or monoclonal antibody against EVs (ncl-entero; clone 5-D8/1) (Novocastra) was used. For detection of EV1, rabbit antiserum against purified EV1 (66) was used. Antibodies against the ER stress markers were obtained from the ER stress antibody sampler kit (Cell Signaling Technologies). Other antibodies were monoclonal (NCL-VIM-V9; Leica Microsystems) and rabbit polyclonal antibody against vimentin (H-84) (Santa Cruz Biotechnology, Inc.), in addition to monoclonal antibody against dsRNA (J2; English & Scientific Consulting). GM130 and PDI antibodies were from Abcam, and G3BP1, PABP, eIF4G, and glyceraldehyde-3-phosphate dehydrogenase (GAPDH) antibodies were from Santa Cruz. Antibody against beta-tubulin was from Cedarlane. Viral protease antibodies have been previously published (67). Antibody against 3D was a kind gift from Antonio Toniolo (Università dell’Insubria, Italy).

### Transfection of poly(I:C).

A549 cells were transfected with poly(I:C) (Santa Cruz) using Lipofectamine 3000. The amount of poly(I:C) was 1 ng/μl or 50 ng/μl, and transfection was carried out according to the instructions of the manufacturer. Cells were fixed with 4% PFA after 2, 4, or 6 h posttransfection. As a control, cells were treated with transfection reagents only with no poly(I:C).

### RT-qPCR.

CVB3-infected A549 cells were freeze-thawed three times and cell debris pelleted down at full speed with a tabletop centrifuge. Viral RNA from the supernatant was extracted according to the instructions of the manufacturer using a QIAamp viral RNA mini kit (Qiagen). Reverse transcription was carried out for positive- or negative-strand RNA using either 1.2 μM antisense (5′-GAAACACGGACACCCAAAGTA) or sense (5′-CGGCCCCTGAATGCGGCTAA) primer, 20 U M-MLV reverse transcriptase (Promega), 4 U RNAsin RNase inhibitor (Promega), and deoxynucleoside triphosphates (Promega). From the reverse transcription reaction mixture (40 μl), 5 μl was taken for PCR, which also contained Sybr green supermix (Bio-Rad) and 600 nM each primer. PCR was performed using a C1000 Touch Thermal cycler (CFX96 real-time system; Bio-Rad), and the amplification steps were 95°C for 10 min, 40 cycles of 95°C for 15 s to 60°C for 1 min, and final melt at 72 to 95°C, with increments of 1°C/5 s. The assay contained three replicates of each sample and also contained negative controls to confirm the specificity of the products.

### Metabolic labeling.

A549 cells were infected with 4.43 × 10^8^ PFU/ml of CVB3. The virus was bound on ice for 1 h, after which the excess virus was washed with PBS. IDPN (1.5%) in DMEM supplemented with 1% FBS was added after ice binding. After IDPN was added, it was present at all steps until the end of the experiment. The infection was allowed to proceed at 37°C for 4 h, after which the low methionine/cysteine medium supplemented with dialyzed 1% FBS was added to cells. After 30 min, 500 μCi/ml of [^35^S]methionine-cysteine was added before a 1-h pulse. Samples were run at 4 to 20% Mini-Protean TGX Stain-Free gel (Bio-Rad), after which the gel was fixed with 30% methanol, 10% acetic acid for 30 min. The gel then was treated with an autoradiography enhancer (Enlightning; PerkinElmer) for 30 min. Finally, the gel was dried at 70°C for 2 h (gel dryer 583; Bio-Rad), and the dried gel was subjected to autoradiography.

### UV-inactivated EV1.

Previous experiments for UV inactivation of picornaviruses (68, 69) were used as a guide for the general settings. Viruses were irradiated with a Sylvania UV-C lamp (UV 8 H, 630 W, Japan) with intensity of 1.8 mW/cm^2^ for 30 s. Lamp intensity was calibrated with a spectrophotometer.

### Neutral red CVB3.

NR-CVB3 was produced in the presence of 10 μg/ml of NR (catalog number 101369; Merck). The virus was released after overnight infection by freeze-thawing the cells and harvested by centrifugation. During the experiment, cells were kept in the dark except for light inactivation, which was for 10 min.

### Crystal violet experiment (CPE).

The cells were washed with PBS to remove the detached cell. Remaining cells were stained with crystal violet stain (0.03%, wt/vol, crystal violet; 2% ethanol; 3% formalin in water). The plate was incubated at room temperature for 10 min and the unbound stain removed. After washes with sterile water, lysis buffer (8.98%, wt/vol, sodium citrate, 125 mM HCl, 47.0% ethanol) was added to the cells, and absorbance was measured from the homogenized solution at a wavelength of 570 nm using a Victor microplate reader.

### Imaging and analysis.

Samples were imaged with an Olympus FV1000-IX81 or Zeiss LSM700 confocal microscope. Appropriate excitation and emission settings were used (405-nm diode laser, 488-nm argon laser, and 543-nm HeNe laser). A UPLSAPO objective (60×; numeric aperture, 1.35) and 20×/0.5 EC Plan-Neofluar objective with resolution of 512 by 512 or 640 by 640 pixels/image were used. Levels for the laser power, detector amplification, and optical sections were optimized for each channel before starting the imaging. The threshold for each channel was adjusted to separate the signal from noise, and data from the images were quantified using a free, open-source software package, BioImageXD (70). In order to quantitate the relative amount of antigen per cell, the total intensity was divided by DAPI-stained nucleus volume or the total intensity of another antigen to gain the ratio of different antigens. For quantification of fluorescent intensities and the relative amount of the immunolabeled antigen, at least 30 cells from three independent experiments were imaged unless otherwise stated. Quantifications were done on single-section images taken from the center of the cell.

### Statistical analysis.

Statistical analysis was performed with GraphPad Prism software. Statistical significance of pairwise differences was determined by Student’s *t* test. All data are presented as means ± standard errors of the means (SEM).
